# Apoptotic Caspases-3 and -7 Cleave Extracellular Domains of Membrane-Bound Proteins from MDA-MB-231 Breast Cancer Cells

**DOI:** 10.3390/ijms26083466

**Published:** 2025-04-08

**Authors:** Eva Vidak, Matej Vizovišek, Nežka Kavčič, Monika Biasizzo, Marko Fonović, Boris Turk

**Affiliations:** 1 Department of Biochemistry and Molecular and Structural Biology, Jožef Stefan Institute, Jamova 39, SI-1000 Ljubljana, Slovenia; evidakijs@gmail.com (E.V.); mvizovisek@gmail.com (M.V.); nezkic@gmail.com (N.K.); marko.fonovic@ijs.si (M.F.); 2 Jožef Stefan International Postgraduate School, Jamova cesta 39, SI-1000 Ljubljana, Slovenia; 3 Faculty of Chemistry and Chemical Technology, University of Ljubljana, Vecna pot 113, SI-1000 Ljubljana, Slovenia

**Keywords:** apoptotic caspases, ectodomain shedding, extracellular activity, cancer cells, membrane-bound proteins

## Abstract

Apoptotic executioner caspases-3 and -7 are the main proteases responsible for the execution of apoptosis. Apoptosis is the main form of programmed cell death involved in organism development and maintenance of homeostasis and is commonly impaired in various pathologies. Predominately an immunologically silent form of cell death, it can become immunogenic upon loss of membrane integrity during progression to secondary necrosis, which mostly occurs when apoptotic bodies are not efficiently cleared by efferocytosis. In cancer, the efferocytic capacity can be overwhelmed following chemotherapeutic treatment, thereby providing an opportunity for the potential extracellular functions of executioner apoptotic caspases in the tumor microenvironment. By triggering apoptosis in Jurkat E6.1 acute T cell leukemia cells, we demonstrated that during progression to secondary necrosis, executioner caspases-3 and -7 can be found in the extracellular space. Furthermore, we showed that extracellularly active caspases-3 and -7 can cleave extracellular domains of membrane-bound proteins from MDA-MB-231 breast cancer cells, a function generally executed in the tumor microenvironment by several extracellular proteases from metalloprotease and cathepsin families. As such, this study provides the evidence for the potential involvement of apoptotic caspases-3 and -7 in extracellular proteolytic networks. Presented mass spectrometry data are available via ProteomeXchange with identifier PXD061399.

## 1. Introduction

Caspases are a family of cysteine proteases that predominantly cleave their substrates after aspartic acid and are mainly involved in the processes of cell death and inflammation [1,2]. Generally divided into apoptotic and inflammatory caspases, they are the driving force behind several types of cell death, among which their proteolytic role predominates in apoptosis and pyroptosis [1,3,4]. Apoptosis, as a major form of programmed cell death crucial for normal development, tissue homeostasis, and the removal of harmful cells that are genetically unstable or aberrantly growing [5,6,7], is initiated by either intracellular or extracellular stimuli and then first mediated by initiator caspases, followed by the main proteolytic action executed by executioner caspases, mainly caspase-3 and -7 [8]. Apoptosis is considered immunologically silent since apoptotic cell-corpses are cleared prior to the loss of plasma membrane integrity, but under certain conditions, progressive loss of plasma membrane integrity can occur, leading to progression to secondary necrosis [9]. Progression to secondary necrosis has been observed in vivo in tumor cells from patients receiving selected chemotherapy, presumably due to insufficient clearance by scavenging cells [10,11]. Additionally, defective efferocytosis of apoptotic cell-corpses has been linked to promotion of inflammation and plaque instability in advanced atherosclerosis [12], further showing that, especially in pathological conditions, apoptotic cells can progress to secondary necrosis. Progression to secondary necrosis renders apoptosis from tolerogenic to immunogenic with intracellular components of the apoptotic machinery being released into the extracellular space [13]. Beyond leakage of intracellular immunostimulatory danger-associated molecular patterns (DAMPs), active caspases could also be released, since extracellular caspase-3-like activity has already been detected both in vitro and in vivo upon apoptosis triggering [14]. Following their activation, executioner caspases have been demonstrated to cleave numerous proteins during apoptosis, primarily those involved in facilitating the process. However, all the studies were focused on intracellular targets [15,16,17,18], and there has been no systematic approach to detect potential extracellular or membrane-anchored targets.

One of the diseases accompanied by aberrant proteolytic activity is cancer, which presents one of the leading causes of premature death [19]. Proteolysis has emerged as a common denominator of diverse mechanisms that accompany cancer hallmarks [20,21], with various proteases being involved at different stages of the disease [22,23,24]. In particular, altered localization of active proteases, and consequently, the corresponding proteolytic activity, can be observed in the tumor microenvironment and is considered particularly relevant in melanoma, glioblastoma, lung cancer, and breast cancer [25]. Among these types of cancer, breast cancer represents the most frequently diagnosed cancer in women and the fifth cause of cancer-related deaths worldwide, thereby warranting special attention [26]. Among the different types of breast cancer, triple-negative breast cancer (TNBC), which accounts for 15–20% of all breast cancer diagnoses [27], is emerging as a clinically more aggressive and very challenging type with worst prognosis and very limited treatment options [28]. The main characteristic of TNBC is the lack of receptors for progesterone and estrogen and the simultaneous lack of overexpression of the receptor tyrosine-protein kinase erbB-2 (HER2) [29], thereby limiting the availability of specific treatment options. Consequently, elucidation of the proteolytic network that accompanies the invasiveness and aggressiveness of TNBC could prove beneficial for the improvement and even development of therapeutic options.

Protease secretion is often responsible for the proteolytic remodeling of the extracellular matrix (ECM) of the tumor microenvironment or for altering the surface and properties of cancer cells through ectodomain shedding, a process mainly attributed to matrix metalloproteases, serine proteases, and cysteine cathepsins [30,31]. Ectodomain shedding can directly influence the properties of both cancer cells as well as other cells present in the tumor microenvironment through modifications of receptor-mediated signaling [25,32,33,34,35], cell–cell contact [36,37,38], and cell adhesion to the components of the ECM [39,40,41]. Additionally, this mechanism could also affect the ability of the immune system to recognize cancer cells as a consequence of the removal of potential recognition signals from the cell surface [42]. In breast cancer, inhibition of receptor shedding has long been studied as a potential therapeutic strategy, with one of best-known drugs, trastuzumab, also inhibiting the shedding of HER2 [43]. Beyond therapy, inhibition of extracellular proteolytic activity in breast cancer, leading to decreased ectodomain shedding, has been proposed as a strategy for overcoming cancer drug resistance [44,45], further emphasizing the importance of the extracellular proteolytic network in breast cancer.

In this study, we showed that apoptotic caspases-3 and -7 can be active in the extracellular milieu and perform limited proteolysis in the extracellular space. We showed that during the progression of apoptosis, the extracellular activity of caspases can be detected mainly from immune cells, often recognized as the main sources of extracellular proteases in the tumor microenvironment [30,46,47], and to a lesser extent from cancer cells. Furthermore, caspase-mediated extracellular proteolysis was observed even in a slightly acidic environment, which is often considered as a characteristic of the tumor microenvironment. Using MDA-MB-231 breast cancer cells, we were able to detect extracellular proteolytic cleavages of ectodomains of membrane-anchored proteins in both physiological and slightly acidic pH environments, showing that apoptotic caspases-3 and -7 can be recognized as a part of the proteolytic network in the case of TNBC. Collectively, the presented evidence opens up the possibility that executioner caspases-3 and -7 could play a role in the extracellular regulation of proteolytic signaling pathways.

## 2. Results

### 2.1. Active Caspases-3 and -7 Can Be Released into the Extracellular Millieu

**Figure 1:**
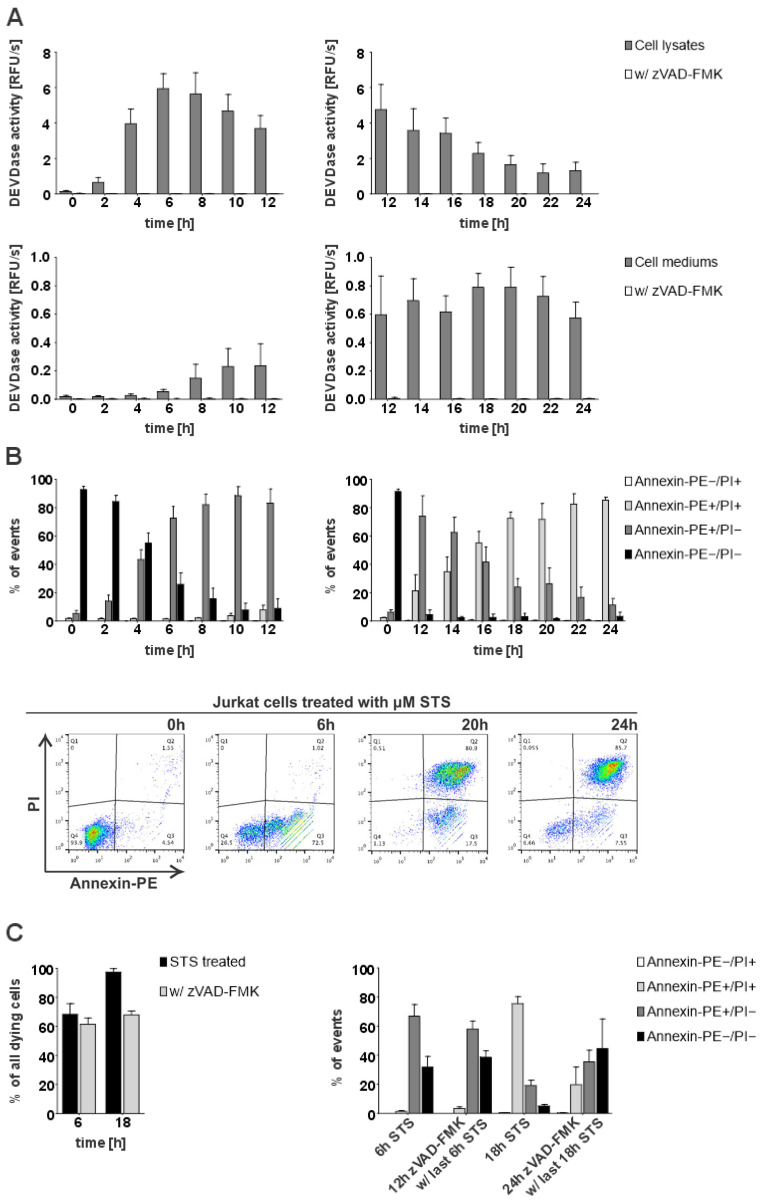
Detection of intracellular and extracellular caspase activity and flow cytometry analysis of STS-triggered apoptosis in Jurkat E6.1 cells. For all time-course experiments, Jurkat E6.1 cells were treated with 0.5 µM staurosporine (STS) or additionally with 25 µM zVAD-FMK. (**A**) DEVDase activity in lysates and cell media following STS treatment. Results are shown for 4 repeated experiments (n = 4) in technical triplicates (n = 3). (**B**) Statistical analysis of flow cytometry measurements depicting the percent of either viable (Annexin-PE−/PI−), early apoptotic (Annexin-PE+/PI−), late apoptotic or secondary necrotic (Annexin-PE+/PI+) or necrotic cells (Annexin-PE−/PI+) detected during four separate experiments (n = 4) and density diagrams of Annexin-PE positive cells (FL1-Height) against PI-positive cells (FL3-Height) present at the representative time points—viable cells (0 h), early apoptosis (6 h), secondary necrosis (20 h) and at the end of the experiment (24 h). (**C**) Cumulative percentages of detected dying cells (either Annexin-PE+/PI−, Annexin-PE−/PI+, or Annexin-PE+/PI+) and analysis of detected stained cells at representative time points for early (6 h) and late apoptosis (18 h) after treatment (n = 2). The data are shown as means ± SD.

To investigate the potential presence and role of active caspases-3 and -7 in the extracellular milieu, we first examined whether caspase activity (DEVD-ase activity) can be detected in the medium of apoptotic cells. For this purpose, we initiated apoptosis in Jurkat E6.1 cells with staurosporine (STS) and checked for the presence of DEVD-ase activity both in cell lysates and cell media (Figure 1A). Our results showed that DEVD-ase activity in cell lysates was highest at earlier time points, reaching peak activity 6 h after the addition of STS and then steadily decreasing during the progression of apoptosis. In contrast, DEVD-ase activity in cell media was not detectable at the early time points, but steadily rose during apoptosis progression, reaching peak activity 20 h post-addition of STS (Figure 1A). However, DEVD-ase activity in the medium remained lower than the activity detected in the lysates.

To elucidate when exactly during the progression of apoptosis extracellular DEVDase activity reached the highest levels, we analyzed the time course of STS-triggered apoptosis in Jurkat E6.1 cells using the Annexin-V/PI staining assay. Our results showed that at the later time points with the highest detected DEVDase activity in cell media, most cells were both Annexin-V- and PI-positive (Figure 1B), suggesting that the presence of extracellular caspase activity coincides with the loss of membrane integrity during the progression of apoptosis to the stage of secondary necrosis. Additionally, we investigated how the inhibition of caspases with pan-caspase inhibitor zVAD-FMK influences the course of STS-triggered apoptosis in Jurkat E6.1 cells. Expectedly, pre-treatment with zVAD-FMK slowed down the course of STS-initiated apoptosis but it did not completely inhibit the observed cell death. Accordingly, only 20% of pre-treated cells were Annexin-V- and PI-positive 18 h post-STS treatment in comparison to around 80% in the absence of zVAD-FMK pre-treatment (Figure 1C). In addition, we compared the activities of extracellular caspases in the Jurkat E6.1 cells with the activities observed in the breast cancer cell line MDA MB-231 and found out that in the latter, they were about twice lower which is in agreement with the reported lower expression levels of caspases in MDA-MB-231 cells as compared to Jurkat E6.1 cells (Figure S1).

**Figure 2:**
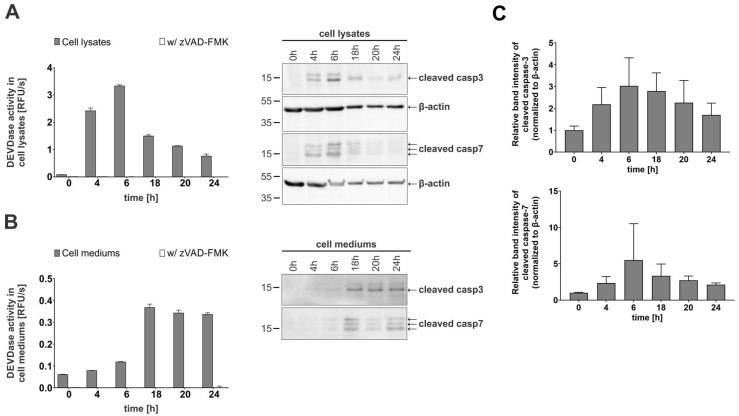
Enzymatic activity and immunological detection of caspases-3 and -7 in lysates and cell media of apoptotic Jurkat E6.1 cells. For all time-course experiments, Jurkat E6.1 cells were treated with 0.5 µM staurosporine (STS). (**A**) DEVDase activity in lysates and corresponding Western blot detection of cleaved caspases-3 and -7. For Western blot detection, β-actin was used as a loading control. (**B**) DEVDase activity in cell media and the corresponding Western blot detection of cleaved caspases-3 and -7. (**C**) Quantification of detected caspases in cell lysates presented as relative intensity of bands normalized to loading control. Three (n = 3) independent repetitions were used to calculate the relative intensity of bands. Kinetic measurements were performed in triplicates (n = 3). Data are presented as means ± SD.

Next, we investigated whether active caspases-3 and -7 are mainly responsible for the observed DEVD-ase activity and could therefore be detected in the cell media and lysates of apoptotic Jurkat E6.1 cells with Western blotting. Stemming from the initial detection of DEVD-ase activity in the lysates and the cell media, we selected time points covering both early apoptosis and late apoptosis or secondary necrosis. The obtained results corresponded with the observed trends for DEVD-ase activity for both lysates (Figure 2A) and cell media (Figure 2B), with cleaved caspases-3 and -7, representing the active forms of both caspases, giving strong signals in the lysates during early apoptosis followed by a steady decline in signal intensity (Figure 2C). In contrast, in the cell media, cleaved caspases-3 and -7 were not detectable during the early apoptosis stage, but only during progression to late apoptosis and secondary necrosis (Figure 2B).

These results show that active caspases-3 and -7 can be found in the extracellular milieu during the progression of apoptotic cells towards the secondary necrotic stage of apoptosis.

### 2.2. Caspase-7 Can Retain Partial Activity in an Acidic Environment

**Figure 3:**
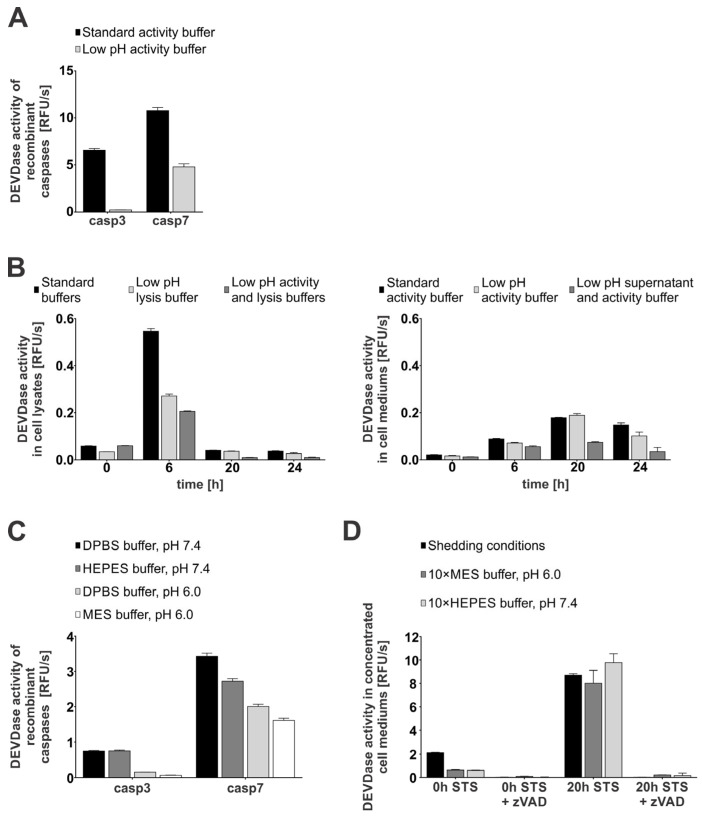
Enzymatic activity of caspases-3 and -7 in a normal (pH 7.4) and acidic (pH 6.0) environment. For DEVDase activity detection, either recombinant caspases (10 nM) were used (**A**,**C**) or apoptosis was induced with STS in Jurkat E6.1 cells and activity was detected in lysates (**B**) and media (**D**). All activity measurements were performed using 20 μM Ac-DEVD-AFC as a substrate (2000:1 ratio) when recombinant caspases were used (**A**,**C**). Jurkat E6.1 cells were treated with 0.5 µM STS at indicated time points. All experiments were performed as technical triplicates (n = 3) and data are presented as means ± SD. (**A**) DEVDase activity of recombinant caspases-3 and -7 in standard (HEPES buffer, pH 7.4) and low-pH (MES buffer, pH 6.0) activity buffer. (**B**) Detection of DEVDase activity in lysates and cell media of STS-treated Jurkat E6.1 cells in normal (pH 7.4) and acidic (pH 6.0) conditions. (**C**) Analysis of DEVDase activity of recombinant caspases-3 and -7 in different buffers (DPBS, HEPES, MES) under normal (pH 7.4) and acidic pH (pH 6.0). (**D**) Detected DEVDase activity in concentrated media from untreated (0 h STS) and secondary necrotic (20 h STS) Jurkat E6.1 cells under shedding conditions.

Next, we investigated whether caspases-3 and -7 remain active in an acidic pH environment. For this, we first measured the DEVDase activity of recombinant caspases-3 and -7 in both slightly acidic (MES buffer, pH 6.0) and neutral pH buffer (HEPES buffer, pH 7.4) supplemented with sucrose and CHAPS to ensure optimal conditions for caspase activity [48]. Our results showed that caspase-3 lost most of its activity in a slightly acidic environment as compared to at a neutral pH, while caspase-7 was less pH-sensitive and retained approximately half of its activity also at an acidic pH (Figure 3A). We next tested whether caspases remain active in cell lysates at acidic pHs using the cell lysates and cell media of STS-treated Jurkat E6.1 cells. As shown, DEVDase activity was observed in both acidic (pH 6.0) and neutral conditions (pH 7.4) (Figure 3B), but was significantly lower at acidic pHs in the case of cell lysates. On the contrary, the activity observed in cell mediums of STS-treated cells remained comparable in both acidic (low-pH activity buffer) and neutral conditions (standard activity buffer).

Next, we assayed the caspase activity in buffers lacking CHAPS to mimic more physiological conditions (Figure 3C). Our results confirmed the trend observed in buffers containing CHAPS and sucrose, with caspase-3 activity being almost undetectable at acidic pH, while caspase-7 activity was less sensitive to pH, with more than half of the detectable activity remaining at acidic pH. To further explore the influence of pH and buffer composition on the extracellular caspase activity, we measured DEVD-ase activity in concentrated media of untreated (0 h STS) and secondary necrotic (20 h STS) Jurkat E6.1 cells (Figure 3D). We adjusted the pH of concentrated supernatants by using concentrated buffers with neutral and acidic pH (Figure S2A). The obtained data correlate well with the observed trends, with the DEVD-ase activity in the pooled concentrated media of the secondary necrotic cells remaining largely retained even after the pH of the media was lowered with the concentrated MES buffer. Additionally, immunological detection showed no significant degradation of caspases-3 and -7 following the addition of concentrated buffers and other components used for shedding conditions (Figure S2B). Moreover, these data suggest that caspase-7 is the main active executioner caspase at acidic pH. In addition, these findings support the possibility of caspase-mediated proteolysis under less favorable conditions, such as those often present in the tumor microenvironment.

### 2.3. Caspases-3 and -7 Can Cleave Extracellular Domains of Membrane-Bound Proteins

**Figure 4:**
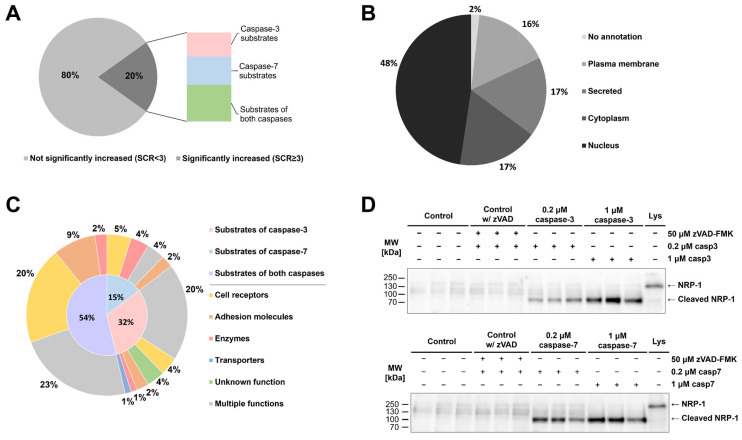
Identification of potential extracellular targets of caspases-3 and -7 and validation of neuropilin-1 cleavage. (**A**) Percentage of identified proteins with significantly increased presence (SCR ≥ 3) of MDA-MB-231 cells in sheddomes upon treatment with both caspases. (**B**) Localization of identified proteins with SCR ≥ 3. (**C**) Division of identified substrates among unique substrates of each investigated caspase and shared substrates, and further division according to the molecular function of identified substrates. (**D**) Validation of caspase-mediated cleavage of the extracellular domain of NRP-1 by caspases-3 and -7 using immunological detection. The experiment was performed in triplicate, but only one experiment is shown.

Having shown that active caspases can be found in the extracellular milieu, we next treated MBA-MB-231 breast cancer cells with recombinant caspases-3 and -7 and performed a mass spectrometry-based identification of cleaved proteins. Since cysteine cathepsins have also been identified as extracellular sheddases [30] and could therefore mask the results, we used broad-spectrum cathepsin inhibitor E-64 in all the experiments. Over 2000 proteins were identified in each experiment; however, the majority were not proteolytically processed. Using a threefold increase in SCR (SCR ≥ 3) between treated and control sample as a threshold, we identified over 600 different proteins, which were cleaved either by only one investigated caspase, 166 proteins in the case of caspase-7 and 141 proteins in the case of caspase-3, or in most cases by both caspases (308 proteins) (Figure 4A). Among the cleaved proteins, nuclear proteins represented almost half of the identified proteins, while membrane-anchored, secreted or cytosolic proteins each representing less than one-fifth of the identified proteins (Figure 4B). As the processing of extracellular domains of membrane-bound proteins is commonly observed in various inflammatory conditions as well as in the tumor microenvironment [49], we next focused on membrane-anchored proteins. In total, 83 potential membrane-anchored caspase targets were found in the sheddomes of both caspases, mostly from the groups of cell receptors and cell adhesion proteins (Figure 4C). Most of the potential substrates were cleaved by both investigated caspases, followed by substrates cleaved solely by caspase-3, and the lowest number of substrates were cleaved only by caspase-7 (Figure 4C). Interestingly, peptide coverage analysis of selected substrates with the highest average SCR for both caspases (Table S1, Figure S3) indicated that caspase-mediated cleavages occur at least 80 residues away from the membrane, contrasting what is typically observed in classical shedding, defined as cleavage within 10 to 35 amino acid residues from the membrane [50].

From the list of potential caspase targets, we selected neuropilin-1 (NRP-1) for the subsequent validation of caspase-mediated extracellular cleavages. Results from immunological detection show that extracellular caspases generate a single cleaved fragment of NRP-1 with a size roughly corresponding to the predicted size of the extracellular domain of NRP-1 (Figure 4D). Additionally, we used Procleave [51], a bioinformatic tool for the prediction of protease-specific substrate cleavage sites, to examine the sequence of the extracellular domain of NRP-1 and found that the two predicted cleavage sites with the highest cleavage probability (Procleave score) correlate with the results from immunological detection (Figure S4A).

### 2.4. Caspases Can Cleave Extracellular Membrane Proteins in Normal and Acidic Environments

**Figure 5:**
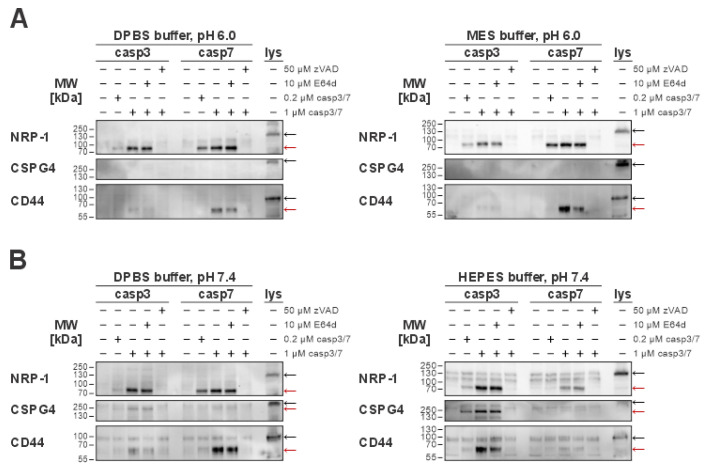
Immunological detection of caspase-mediated cleavages of extracellular proteins in normal (pH 7.4) and acidic (pH 6.0) environments. For the detection of extracellular cleavages, MDA-MB-231 cells were used. Red arrows indicate bands representing cleaved fragments, while black arrows indicate full-length proteins present in total cell lysates. (**A**) Detected caspase-3 or -7 extracellular cleavages of the selected targets (NRP-1, CD44, CSPG4) in an acidic (pH 6.0) environment in either DPBS or MES buffer. (**B**) Detection of caspase extracellular cleavages in a normal (pH 7.4) environment using either DPBS or HEPES buffer. NRP-1, neuropilin-1; CD44, CD44 antigen; CSPG4, chondroitin sulfate proteoglycan 4.

Next, we explored how the change in pH affects caspase-mediated extracellular cleavages of membrane-bound proteins. Therefore, we treated MDA-MB-231 cells with recombinant caspases-3 and -7 with neutral (pH 7.4) and slightly acidic (pH 6.0) buffers and checked whether we can detect caspase-generated extracellular cleavage products of target proteins (Figure 5A,B). In order to avoid interreference with cysteine cathepsins, we incubated all cells with broad-spectrum cysteine cathepsin inhibitor E-64d.

First, we focused on NRP-1 cleavages, since these have already been detected in sheddomes used in mass spectrometry (MS) analysis (Figure 3B). We confirmed that both caspases can cleave the extracellular domain of NRP-1 in all tested conditions (Figure 5). As the second target, we selected chondroitin sulfate proteoglycan 4 (CSPG4), because the MS results suggested that it could be a unique substrate of caspase-3. Expectedly, no cleavages of CSPG4 were detected under an acidic pH (Figure 5A). This agrees with both the MS results, identifying CSPG4 as a unique caspase-3 target, and the enzyme kinetics analysis results, showing almost complete loss of caspase-3 activity in acidic pH (Figure 3A,C). At neutral pH, our data suggest that the buffer environment itself could play a role in the extracellular caspase-3-mediated cleavage of CSPG4, with the detection of the cleaved fragment corresponding to the approximate size of the extracellular domain of CSPG4 being more prominent when using HEPES buffer in comparison to DPBS (Figure 5B). Finally, as the last target to investigate, we selected CD44 antigen (CD44). While CD44 was not identified as an extracellular target of caspases by our MS analysis, it is otherwise a common target of other sheddases in the tumor microenvironment [30,52]. Interestingly, our experiments showed that caspases-3 and -7 can cleave CD44 at a concentration of 1 µM for both caspases, with the size of the detected fragment matching the approximate predicted size of its extracellular domain. However, at acidic pH, only caspase-7-mediated cleavage was detected (Figure 5A), whereas at neutral pH both caspases cleaved it. In addition, the buffer system itself seems to impact the cleavage, with more pronounced cleavage of caspase-7 in the DPBS buffer and caspase-3 in the HEPES buffer (Figure 5B).

For both CSPG4 and CD44, we also performed an in silico prediction of possible cleavage sites using the Procleave software (Figure S4). In the case of CSPG4, the predicted cleavage sites with the highest probability score for caspase-3-mediated cleavage correlated well with the observed Western blot results (Figure S4B), while a similar correlation was not observed for CD44, with the predicted size of the cleaved fragment differing from the results of Western blot analysis (Figure S4C).

Taking everything into consideration, caspases-3 and -7 can cleave extracellular domains of membrane-bound proteins at both normal and acidic pH, with NRP-1 being the most prominent target.

### 2.5. Caspase- and Cathepsin-Mediated Shedding Show Different Cleavage Patterns

Since the proteins released from the cell following membrane damage could also be lysosomal cathepsins, among other proteins, we checked how the caspase-mediated shedding here compares to cathepsin-mediated shedding previously reported for cathepsins L and S [30].

**Figure 6:**
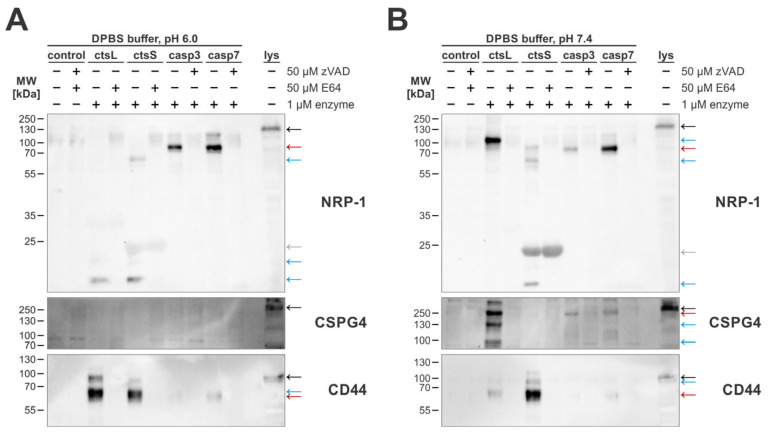
Western blot detection of extracellular cleavages of selected targets following treatment by cathepsins L and S and caspases-3 and -7. For all experiments, MDA-MB-231 cells were used. Cleavages were detected in DPBS buffer with pH 6.0 (**A**) and pH 7.4 (**B**). Red arrows indicate bands representing cleaved fragments produced by caspase-mediated cleavage, black arrows indicate full-length proteins as present in total cell lysates, and blue arrows indicate fragments specific for cathepsin cleavages. Additionally, the gray arrow indicates a non-specific band likely belonging to active cathepsin S.

For this, we treated MDA-MB-231 cells with 1 µM recombinant enzymes using buffers with an acidic pH (Figure 6A), which is ideal for cathepsin activity, and with a neutral pH (Figure 6B), which is best suited for caspase activity. The sheddomes isolated in this experiment were used to detect cleavages of the three tested extracellular caspase substrates—NRP-1, CSPG4, and CD44. For all three selected targets, we were able to show that cathepsins cleave the extracellular domain at multiple sites, generating several cleaved fragments, while caspases generated only a single cleavage fragment. Additionally, our results suggest that apoptotic caspases-3 and -7 and cysteine cathepsins L and S cleave selected targets at different cleavage sites, since we detected several enzyme-specific fragments. Among the three investigated targets, the highest degree of similarity in detected cleavage patterns could be observed for CD44, while cleavage patterns for NRP-1 and CSPG4 showed little overlap. Additionally, in the case of CSPG4, the results suggest that it could potentially be cleaved only at a neutral pH, since no distinct cleaved fragments were detected in the sheddomes produced under acidic conditions.

## 3. Discussion

Elucidation of the biological roles of the caspases extending beyond apoptosis has received increased interest recently [53,54,55,56] and could provide new insights into their mechanisms of action both in physiological conditions as well as in pathologies, which could lead into new directions for future disease management. Here, we have shown that active apoptotic caspases-3 and -7 could be released into the extracellular space during apoptosis and we have performed limited proteolysis on extracellular domains of membrane-bound proteins. This opens an important niche for caspase action beyond apoptosis, since proteolysis of extracellular proteins, such as adhesion molecules or cell surface receptors, is of major importance for the regulation of cell signaling and other functions of cells, especially in the context of cancer [23,57,58,59].

The extracellular presence of active proteases is commonly associated with limited proteolysis of ECM components, various cytokines, endogenous inhibitors, and extracellular domains of membrane-bound proteins, the later termed ectodomain shedding [22,30,57], and it can be often observed in the tumor microenvironment. Although caspases are known to cleave a number of proteins during apoptosis, there has been no evidence that they would be able to cleave extracellular proteins, in particular those inserted into the membrane. Since limited proteolysis in the extracellular space is commonly associated with cancer, we used cancer and immune cells to investigate the potential extracellular roles of apoptotic caspases. Immune cells are often recognized as one of the main sources of extracellular proteases in the heterogenic tumor microenvironment [30,46,47]. Therefore, we selected Jurkat E6.1 cells to investigate whether active executioner caspases could be released in the extracellular space. Due to the similar expression level of both main executioner caspases-3 and -7, Jurkat E6.1 cells enable detection of enzymatic activity independently of intrinsic differences in expression levels of caspases, present in MDA-MB-231 cells (Figure S1B). Using immune cells as a model, we detected extracellular caspases-3 and -7, consistent with an earlier study, which showed the presence of extracellular caspase-3-like activity both in vitro as well as in vivo and even suggested it as a potential biomarker for monitoring excessive apoptotic organ damage, the success of chemotherapeutic treatment and organ transplant survival [14]. Moreover, previous studies implicating apoptotic caspases in extracellular proteolysis have shown that active caspase-3 can be packed in secretory compartments of viable mast cells [60] and, following secretion in a granzyme B-dependent manner, can cleave cytokine IL-33 in the extracellular milieu [61]. In our case, the presence of processed active caspases in the media coincided with the loss of plasma membrane integrity during the progression to secondary necrosis, suggesting that caspases are not actively secreted as a part of the apoptotic program, but rather passively released when apoptotic cells spontaneously progress from apoptosis to secondary necrosis, which can most commonly be observed when phagocytic efferocytosis is either impaired or overwhelmed [13,62]. Focusing on breast cancer, it has also been shown that, in addition to spontaneous progression, accelerated progression can be present as a result of chemotherapeutic treatment, which triggers the progression of apoptosis to secondary necrotic or pyroptotic-like cell death in the tumor microenvironment through the caspase-3-mediated cleavage of GSDME [11,63]. Accelerated progression via the cleavage of GSDME has been observed in various breast cancer cells [64,65], but, in general, it does appear to be quite cell-line specific [66]. Nevertheless, the observed immunogenic apoptotic cell death in the tumor microenvironment offers further grounds for the potential extracellular roles of active apoptotic caspases.

An important factor in the tumor microenvironment that could limit the extracellular activity of caspases-3 and -7 is the acidic pH often observed in this environment [67]. The initial characterization of the enzymatic activity of recombinant caspases revealed a relatively narrow pH range in which they remain active [48], suggesting that in an acidic environment, they would rapidly lose their proteolytic activity. However, caspase-7 retained substantial activity in an acidic pH, in contrast to caspase-3. Comparable extracellular enzymatic activity observed in both pH environments suggests that when released in the acidic environment caspase-7, and not caspase-3, most probably acts as the predominant active caspase, whereas caspase-3 appears to be the main executioner caspase at neutral pH (Figure 3). Retention of caspase activity even below their reported pH optimum is in line with the observation that apoptosis is often accompanied by changes that lead to a lowering of the cytosolic pH [68]. This acidification of the cytosol has been proposed as a modulator of caspase activation, especially cytochrome c-dependent caspase activation, which is higher at a acidic pH and lower at a neutral pH [69]. Furthermore, in the context of cancer, the acidity of the tumor microenvironment could further impact the extracellular presence of caspases, since it has been shown that an acidic pH could lead to the bursting of tumor-shed membrane vesicles [70]. This opens up the possibility for the additional release of active caspases that could initially be packed inside such vesicles or even apoptotic bodies.

Once present in the extracellular space, active caspases seem to be able to process numerous substrates. While a great number of intracellular substrates, mostly related to normal apoptosis, have been identified before [71], there has been no evidence of extracellular substrates. Therefore, we aimed to identify potential extracellular substrates, focusing on a group of membrane-anchored substrates that are known for their role in cell–cell attachment and cellular signaling, processes often impaired in various hallmarks of cancer [20,21]. Using the breast cancer cell line MDA-MB-231 as a model, since in breast cancer, shedding of cell surface proteins has been observed in various studies [32,72], we identified over 80 potential caspase substrates with mass spectrometry-based proteomics (Figure 4). Among the identified potential caspase substrates, NRP-1 appeared as the most prominent target due to the high number of detected peptides and high SCR upon treatment with both caspases-3 and -7. Combined with the confirmation that a caspase-generated fragment of NRP-1 could be detected in the caspase sheddomes (Figure 4D), this provides the first level of evidence that apoptotic caspases could act as sheddases when released into the extracellular space, with roles beyond apoptotic signaling.

Encouraged by the apparent retention of caspase activity even in a sub-optimal pH environment when using both recombinant caspases and media collected from apoptotic immune Jurkat E6.1 cells (Figure 3), we then investigated whether the change in pH impacts the detection of extracellular caspase-mediated cleavages of membrane-bound proteins from breast cancer cells even beyond the most prominently observed cleavage of NRP-1. By exogenously treating MDA-MB-231 breast cancer cells with recombinant caspases-3 and -7 in different pH environments, we have shown that caspases-3 and -7 can cleave NRP-1 and CD44 in both normal and acidic pH, while CSPG4, which was identified as a unique caspase-3 substrate, was expectedly cleaved only in a neutral-pH environment (Figure 5). Interestingly, we detected extracellular caspase-mediated cleavages of CD44, despite initially not identifying it as a substrate of caspases due to a non-significant increase in SCR upon treatment with recombinant caspases. This lack of proteomic identification could be explained by the observed dependency of caspase-mediated CD44 cleavage on the buffer environment used for the treatment (Figure 5).

Since many identified extracellular substrates overlapped with previously identified substrates of cysteine cathepsins [30], we investigated whether caspases and cathepsins generate different cleavage patterns of the extracellular domains of selected targets. We found that cathepsin-mediated cleavages often produce more than one fragment, pointing towards not only ectodomain shedding but also further proteolytic processing and degradation of the extracellular domains of membrane-bound proteins (Figure 6). On the contrary, caspase-mediated cleavage of the three selected substrates resulted in the detection of a single cleaved fragment that approximately matched the size of the complete extracellular domain for all three investigated targets. This difference in observed cleavage patterns is most likely due to the more stringent substrate specificity of caspases [73,74] as compared to more promiscuous cysteine cathepsins [75,76,77,78]. The less stringent substrate specificity of cysteine cathepsins could also be a reason for the lack of detected cathepsin-produced fragments of CSPG4 in a slightly acidic environment. Even though cathepsin L is irreversibly inactivated under a neutral pH [79], we were able to detect cathepsin L-generated fragments of CSPG4 in the neutral pH environment, suggesting that CSPG4 is a target of cathepsin L Figure 6B). Therefore, the lack of any distinct fragments produced by both of the tested cathepsins in a slightly acidic environment could be either due to a complete lack of shedding or due to the complete degradation of the released extracellular domain. Overall, the comparison of caspase- and cathepsin-mediated shedding indicates that caspases are weaker sheddases than cysteine cathepsins, with the potential exemption being NRP-1, which, among the three validated substrates, was the one most efficiently cleaved by caspases.

How would caspases act as sheddases in a physiological setting? Once released, they can clearly cleave several proteins; however, it is much more likely that there is a concerted action of many proteases released or even secreted into the extracellular milieu upon cellular damage or during cancer progression. While caspases surely contribute to the total proteolysis observed in the tumor microenvironment, it is likely that other proteases, which can be found at such sites at a much higher levels, would carry out the bulk of the proteolytic processing. With this study, we therefore provide the first evidence that apoptotic caspases could be recognized as a part of the extracellular proteolytic network. Despite being tightly regulated and recognized as almost exclusively intracellular proteases, we have shown that under specific conditions, they can be detected in the extracellular space where they can retain their proteolytic activity even in sub-optimal conditions and cleave extracellular domains of membrane-bound proteins. This opens up an interesting possibility to consider apoptotic caspases as sheddases capable of modifying cell properties and cell signaling, especially in the tumor microenvironment, either alone or in concert with other proteases.

## 4. Materials and Methods

### 4.1. Reagents

The commercial reagents or kits used in this study were Annexin V-Phycoerythrin (PE) Recombinant Protein (BM306PE, Affymetrix, Santa Clara, CA, USA), Bio-Rad Protein Assay Dye Reagent Concentrate (5000006, Bio-Rad, Hercules, CA, USA), protease inhibitor cocktail (PIC) (P8340, Sigma Aldrich, St. Louis, MO, USA), eBioscience™ Binding Buffer for Annexin V (BMS500BB, Thermo Scientific, Waltham, MA, USA), ECL™ Western blotting Reagents (RPN2106, Cytiva, Marlborough, MA, USA) and PageRuler™ Plus Prestained Protein Ladder (#26619, Thermo Scientific, Waltham, MA, USA). For enzyme kinetics, substrate Ac-DEVD-AFC (#4027914, Bachem, Torrance, CA, USA) and inhibitor zVAD-FMK (#4026965, Bachem) were purchased from Bachem, while cathepsin inhibitors E-64 (#4096, Peptide Institute, Osaka, Japan) and E-64d (#4321-v, Peptide Institute, Osaka, Japan) were purchased from Peptide Institute (Osaka, Japan).

Primary antibodies used for detection were polyclonal rabbit antibodies against cleaved caspase-3 (#9661, Cell Signaling, Danvers, MA, USA), polyclonal rabbit antibodies against cleaved caspase-7 (#9491, Cell Signaling, Danvers, MA, USA), polyclonal rabbit antibodies against β-actin (A2066, Sigma Aldrich, St. Louis, MO, USA), polyclonal sheep antibodies against human neuropilin-1 (#AF3870, R&D Systems, Minneapolis, MN, USA), monoclonal mouse antibodies against human CD44 (#3570, Cell Signaling, Danvers, MA, USA) and rabbit monoclonal antibodies against human CSPG4 (#43916, Cell Signaling, Danvers, MA, USA). As secondary antibodies, HRP-labeled polyclonal goat anti-rabbit (#111-035-045, Jackson ImmunoResearch, Cambridge, UK), rabbit anti-sheep (#313-035-045, Jackson ImmunoResearch, Cambridge, UK), and goat anti-mouse antibodies (#115-035-068, Jackson ImmunoResearch, Cambridge, UK) were used.

### 4.2. Recombinant Enzymes Preparation

Human caspases-3 and -7 [80] and human cathepsins S and L [81] were prepared and purified as previously described, and their active concentration was determined by active site titration [48,82,83].

### 4.3. Cell Culture

MDA-MB-231 breast cancer cells (HTB-26, ATCC, Manassas, VA, USA) were grown in DMEM media (D6429, Sigma Aldrich, St. Louis, MO, USA) supplemented with 10% normal fetal bovine serum (F7524, Sigma Aldrich, St. Louis, MO, USA), 1% glutamine (G7513, Sigma Aldrich, St. Louis, MO, USA) and 1% penicillin/streptomycin (P4333, Sigma Aldrich, St. Louis, MO, USA). The Jurkat E6.1 lymphoblastoid cell line (88042803, ECACC, Salisbury, UK) was grown in RPMI-1640 medium (R5158, Sigma Aldrich, St. Louis, MO, USA), completed with 10% heat-inactivated fetal bovine serum (F9665, Sigma Aldrich, St. Louis, MO, USA), 1% glutamine, and 1% penicillin/streptomycin. Both cell lines were grown in a controlled atmosphere at 37 °C and 5% CO_2_.

### 4.4. Induction of Apoptosis

Jurkat E6.1 cells were seeded at a cell density of 1 × 10^6^ cells/mL in serum-free RPMI-1640 medium and at selected time points treated with 0.5 µM STS (HY-15141, MedChemExpress, Monmouth Junction, NJ, USA). Where indicated, cells were additionally pre-treated with 25 µM of pan-caspase inhibitor zVAD-FMK (benzyloxycarbonyl-Val-Ala-Asp-fluoromethylketone). In the case of MDA-MB-231 cells, 0.5 × 10^6^ cells/well were seeded in a 6-well plate and after reaching confluency, apoptosis was initiated at selected time points by the addition of 0.5 µM STS.

### 4.5. Flow Cytometry

Quantification of apoptosis was performed with Annexin V and Propidium Iodide (PI) staining as previously described [84]. Following STS treatment, cells were pelleted from the cell suspension and stained using 2 µL of Annexin V-PE conjugate and 100 µg of PI for each stained sample. Prepared samples were analyzed with a FACSCalibur flow cytometer (BD Biosciences, Franklin Lakes, NJ, USA). Afterward, compensation of acquired fluorescence intensity plots and density plots was performed with FlowJo software v10 (BD Life Sciences, Franklin Lakes, NJ, USA) to enable discrimination between populations of viable cells (Annexin V-/PI-), early apoptotic cells (Annexin V+/PI-), late apoptotic or secondary necrotic cells (Annexin V+/PI+) and necrotic cells (Annexin V-/PI+).

### 4.6. Preparation of Cell Media and Lysates of STS-Treated Jurkat E6.1 Cells

Cell media were first cleared of cells by centrifugation at room temperature (RT) and 300× *g* for 5 min, followed by additional centrifugation at 4 °C and 13.000× *g* for 15 min. Prepared cell media were either used immediately or stored at −80 °C.

Cell pellets remaining after initial centrifugation were resuspended in lysis buffer (25 mM HEPES, pH 7.5, 5 mM MgCl_2_, 1 mM EDTA, 0.1 (*v*/*v*)% Triton-X-100, 0.5 (*v*/*v*)% NP-40, 0.1 (*v*/*v*)% CHAPS, 1 (*v*/*v*)% PIC), or where indicated, low-pH lysis buffer (25 mM MES, pH 6.0, 5 mM MgCl2, 1 mM EDTA, 0.1 (*v*/*v*)% Triton-X-100, 0.5 (*v*/*v*)% NP-40, 0.1 (*v*/*v*)% CHAPS, 1 (*v*/*v*)% PIC), briefly vortexed and shaken on ice for 45 min to ensure cell lysis. Next, the prepared cell suspensions were again centrifuged at 4 °C and 13.000× *g* for 15 min and supernatants representing cell lysates were collected and either used immediately or stored at −80 °C.

### 4.7. Preparation of Concentrated Cell Media from STS-Treated Jurkat E6.1 Cells

Concentrated media were cleared of cells by initial centrifugation of the cell suspension at RT and 300× *g* for 5 min followed by filtration of the cell media through a 0.2 µm filter. Filtered cell media were concentrated approximately a hundred times by centrifugation at 4 °C and 2.500× *g* using 3K Amicon^®^ Ultra 15 mL centrifugal filters (UFC900324, Merck, Darmstadt, Germany) and stored at −80 °C.

### 4.8. Enzyme Kinetics

Caspase activity was measured by the cleavage of caspase-specific fluorogenic substrate Ac-DEVD-AFC (acetyl-Asp-Glu-Val-Asp-7-amino-4-trifluoromethylcoumarin) using an Infinite M1000 Pro (Tecan, Männedorf, Switzerland) microplate reader with excitation and emission wavelengths set at 400 and 510 nm, respectively. The acquired data were analyzed using GraphPad Prism v6 software (GraphPad Software, La Jolla, CA, USA) with the linear portion of the hydrolysis curve being used in all cases. For all the measurements, a 20 µM final concentration of Ac-DEVD-AFC was used, and, unless stated otherwise, substrate hydrolysis was measured for 30 min.

For the detection of extracellular and intracellular caspase activity, cell media and lysates of STS-treated Jurkat E6.1 cells were mixed with either caspase activity buffer (50 mM HEPES, pH 7.4, 1 (*w*/*v*)% sucrose, 0.1 (*v*/*v*)% CHAPS, 10 mM DTT, 10 μM E-64) or low-pH caspase activity buffer (50 mM MES, pH 6.0, 1 (*w*/*v*)% sucrose, 0.1 (*v*/*v*)% CHAPS, 10 mM DTT, 10 μM E-64) where indicated, with equal volumes being used for each selected time point. Where indicated, the pH of the cell media was additionally adjusted with HCl to pH 6.0. In parallel, pan-caspase inhibitor zVAD-FMK was added to the cell lysates and media to a final concentration of 25 µM. Prepared mixtures were incubated for 1 h at 37 °C before the addition of Ac-DEVD-AFC substrate and detection of substrate hydrolysis.

Evaluation of caspase activity in different pH and buffers was performed using both recombinant caspases-3 and -7 as well as the concentrated medium from apoptotic STS-treated Jurkat E6.1 cells. Selected buffers (Dulbecco’s phosphate-buffered saline (DPBS), pH 7.4 and 6.0, MES buffer, pH 6.0 and HEPES buffer, pH 7.4) were supplemented with 1 mM DTT, 1 mM EDTA, 0.5 mM PMSF and 25 µM E-64, these being later termed shedding conditions. Recombinant caspases-3 and -7 were diluted to a final concentration of 10 nM and their activity was evaluated both in standard- and low-pH caspase activity buffers as well as under shedding conditions in selected buffers. Caspase activity in the concentrated medium was measured under shedding conditions by mixing the pooled concentrated media with selected buffer (10 × MES and 10 × HEPES) in a 9-to-1 ratio. In parallel, pan-caspase inhibitor zVAD-FMK was added to the final concentration of 25 µM. Prepared mixtures were incubated for 1 h at 37 °C before the measurement of kinetic activity.

### 4.9. Immunodetection of Caspases with Western Blotting

Equal volumes of cell media and cell lysates were used for each time point. Media were precipitated with 10% trichloroacetic acid (TCA) before resolution by SDS-PAGE (sodium dodecylsulfate–polyacrylamide gel electrophoresis) followed by Western blot using Western blotting buffer (25 mM Tris, 192 mM glycine) supplemented with 20 (*v*/*v*)% methanol. Additionally, concentrated cell media mixed with selected buffers were also analyzed by Western blotting as described. Before Western blot analysis, the pH of the samples was confirmed using the InLab Micro pH electrode (Mettler Toledo, Columbus, OH, USA). To prevent non-specific binding, membranes were gently shaken at RT in 5% skimmed milk in Tris-Buffered Solution (TBS) with added 0.05 (*w*/*v*)% Tween-20 (TBST buffer), followed by overnight incubation with primary antibodies at 4 °C. For detection, the membranes were incubated for 1 h with horseradish peroxidase (HRP)-labeled secondary antibodies and then specific binding was visualized on G:BOX Chemi XR (Syngene, Cambridge, UK) using ECL reagent (#RPN2106, Cytiva, Marlborough, MA, USA) according to the manufacturer’s instructions.

Acquired images were analyzed using ImageJ software version 1.54p, and the intensity of bands representing cleaved caspases-3 and -7 in cell lysates was quantified and normalized against the loading controls. For bands observed in cell media, relative intensities were calculated with the time point without exposure to STS (0 h), as a control intensity, and results were presented as fold of control (Figure S5).

### 4.10. Treatment of MDA-MB-231 Cells with Recombinant Caspases for Target Identification

MDA-MB-231 breast cancer cells were cultured to confluency before treatment with recombinant enzymes. Confluent cells were washed two times with DPBS with Ca and Mg (D8662, Sigma Aldrich, St. Louis, MO, USA) and detached with Hank’s based enzyme-free cell dissociation solution (S-004-B, Millipore, Burlington, MA, USA). The detached cells were pelleted by centrifugation for 4 min at RT and 400× *g* and then resuspended in DPBS buffer.

For MS analysis, resuspended cell pellets were prepared as previously described [30]. Briefly, cells were divided into 4 aliquots, each containing approximately 30 × 10^6^ cells, and then resuspended in control buffer (DPBS buffer, pH 7.4, 1 mM DTT, 1 mM EDTA, 0.5 mM PMSF), control buffer with added zVAD-FMK (50 μM) and E-64 (50 μM) and control buffer containing either low (0,2 μM) or high (1 μM) active concentrations of caspase-3 or -7. Following incubation at 37 °C for 1 h, the supernatants were collected after sequential centrifugation first at RT and 500× *g* for 5 min and then at 4 °C and 14,000× *g* for 5 min. Any residual caspase activity in supernatants was blocked with pan-caspase inhibitor zVAD-FMK (50 μM). Prepared supernatants containing caspase-produced sheddomes were stored at −80 °C until further use.

Additionally, MDA-MB-231 breast cancer cells were incubated with caspases for the validation of the most prominent MS-identified targets with immunodetection. Confluent cells were detached as described and divided into 4 aliquots each containing approximately 5 × 10^6^ cells. Cell pellets were resuspended in control buffer (DPBS buffer, pH 7.4, 1 mM DTT, 1 mM EDTA, 0.5 mM PMSF, 25 µM E-64), in control buffer with added zVAD-FMK (50 μM), and a 0.2 μM concentration of selected caspases, and in control buffer containing either a 0.2 μM or 1 μM active concentration of selected caspases. Afterward, prepared samples were incubated and supernatants were collected and prepared for SDS-PAGE analysis as described.

### 4.11. Mass Spectrometry Analysis

Supernatants containing the caspase sheddomes were concentrated approximately five times using 3K Amicon^®^ Ultra 0.5 mL centrifugal filters (UFC500396, Merck, Darmstadt, Germany), and one-third of each concentrated supernatant was loaded onto 12.5% SDS-PAGE Precast gel (#58509, Lonza, Basel, Switzerland). The SDS-PAGE gels were then prepared for mass spectrometry analysis as previously described [30].

Analysis of extracted peptides from SDS-PAGE gels was performed by liquid chromatography with tandem mass spectrometry (LC-MS/MS) using EASY-nanoLC II HPLC unit (Thermo Fischer Scientific, Waltham, MA, USA) coupled to an Orbitrap LTQ Velos mass spectrometer (Thermo Fischer Scientific, Waltham, MA, USA) and operated via Xcalibur software version 2.2 SP1.48 (Thermo Fischer Scientific, Waltham, MA, USA). Parameters for LC-MS/MS data acquisition were set as previously described [30]. Briefly, peptide samples were loaded on a C18 Proxeon EASY-Colum^TM^ (Thermo Fischer Scientific, Waltham, MA, USA), separated using C18 PicoFrit^TM^ AQUASIL analytical column (New Objective, St. Littleton, MA, USA) and finally eluted with a linear gradient of 0.1% formic acid in acetonitrile (90 min, solvent gradient of 5–50%, flow rate of 300 nL/min). Acquired data were analyzed using MaxQuant proteomics software (version 1.5.2.8, Max Planck Institute, Munich, Germany) with an embedded Andromeda search engine [85,86]. Searches were performed against the canonical database of the human reference proteome derived from the UniProt database (UniProtKB, Homo Sapiens, canonical database containing 16,670 entries, released in July 2014) with the exclusion of protein isoforms. Further restrictions for cleavage specificity, modifications, precursor and mass fragment tolerance, and false discovery rate were set as previously described [30]. All the protein groups with at least two identified peptides in the MaxQuant search were considered a positive identification and their relative abundance in the treated sample as compared to the control was calculated using the Perseus software v.1.6.0.7 [87]. Spectral counting of their razor and unique peptides was used as a semiquantitative measure of protein abundance in sheddomes collected from caspase-treated cells [88], with the threshold for relative abundance in treated samples set as at least a threefold increase in the spectral counts for selected protein. Additionally, only proteins with known localization at the cell membrane were considered potential extracellular targets of caspases. Identified proteins with the highest average SCR for both caspases were further analyzed with the Draw Map tool, which enabled the visualization of protein sequence coverage with peptides detected by LC-MS/MS [89].

### 4.12. Treatment with Recombinant Caspases and Cathepsins in Different Conditions

MDA-MB-231 breast cancer cells were cultured and detached as described, and approximately 1 × 10^6^ cells were used for each tested condition. Cell pellets were resuspended in four different buffers (DPBS, pH 7.4; DPBS, pH 6.0; HEPES buffer, pH 7.4, MES buffer, pH 6.0) for treatment with recombinant caspases-3 and -7 and in DPBS buffers with acidic and neutral pH for treatment with both caspases and with cathepsins L and S. Treatment with just caspases was performed under standard shedding conditions, while during treatment with both caspases and cathepsins, E-64 was omitted and added only where indicated. Additionally, for caspase treatment, one-fifth of the cells were pre-treated overnight with 10 µM E-64d, and after detachment, 10 µM E-64d was also added to the resuspension buffer where indicated.

For treatment with recombinant caspases, cells were divided into five aliquots corresponding to the control sample, the sample treated with 0.2 µM and 1 µM concentrations of the selected caspase, the E-64d-inhibited sample treated with a 1 µM concentration of the selected caspase and the zVAD-FMK-inhibited sample also treated with 1 µM of caspase. The buffer for the zVAD-FMK-inhibited sample was supplemented with both the inhibitor and selected caspase approximately 1 h before cell resuspension, while to the buffers used for other samples, caspases were added just before resuspension. In the case of combined treatment with both caspases and cathepsins, cells were divided into ten aliquots, with one corresponding to the control (selected buffer), one to the inhibited control (selected buffer with 50 µM E-64 and 50 µM zVAD-FMK), four to treatment with the 1 µM selected recombinant enzyme and four to treatment with the inhibited enzyme (1 µM of selected recombinant enzyme and 50 µM of appropriate inhibitor). Resuspension buffer for high-concentration treatment with inhibitor was supplemented with both the selected enzymes (caspase-3 and -7 or cathepsins L and S) and appropriate inhibitor (zVAD-FMK for caspases and E-64 for cathepsins) approximately 1 h before use while resuspension buffers for other conditions were supplemented with selected recombinant enzyme immediately before resuspension.

Resuspended cells were incubated at 37 °C for 1 h and supernatants containing sheddomes were collected. Any residual enzymatic activity in the collected supernatants was blocked by the addition of E-64 (50 µM) and zVAD-FMK (50 µM). Collected supernatants were mixed with acetone and precipitated as previously described [90,91]. Dry pellets containing precipitated proteins were prepared for SDS-PAGE.

### 4.13. Immunodetection of Extracellular Targets with Western Blotting

For the immunodetection of cleavages of extracellular targets concentrated supernatants prepared for LC-MS/MS analysis and acetone-precipitated supernatants were used. Concentrated supernatants used for LC-MS/MS analysis were resolved on SDS-PAGE and electrotransferred as described, while precipitated supernatants were electrotransferred using Western blotting buffer supplemented with 10 (*v*/*v*)% methanol.

Additionally, a control containing approximately 200 µg of untreated MDA-MB-231 cell lysate was loaded each time. The cell lysate was prepared by resuspending enzyme-free detached cells in cell lysis buffer (50 mM Tris-HCl, pH 7.4, 100 mM NaCl, 1 mM EDTA, 1 (*v*/*v*)% NP-40, 0.1 (*v*/*v*)% SDS, 0.1 (*v*/*v*)% deoxycholate, 1 (*v*/*v*)% PIC) and performing mechanical cell lysis using a 26 G syringe needle (#303800, BD Microlance, Franklin Lakes, NJ, USA). Afterwards, cell suspensions were incubated on ice for 30 min with vigorous shaking. Prepared lysates were cleared of insoluble material by centrifugation at 4 °C and 16.000× *g* for 10 min and then the concentration of proteins in the lysates was determined using a Bradford protein assay (Bio-Rad, Hercules, CA, USA).

Acquired Western blotting images were analyzed using ImageJ software and the relative intensity of observed bands was calculated as compared to the intensity of bands in the control sample containing sheddomes after the treatment of cells with solely the buffer. A graphical presentation of the relative intensity of observed bands presented as a fold of the control is available in the Supplementary Materials (Figure S6).

## Data Availability

The original contributions presented in this study are included in the article and Supplementary Material. The mass spectrometry proteomics data have been deposited to the ProteomeXchange Consortium via the PRIDE [92,93] partner repository with the dataset identifier PXD061399. Further inquiries can be directed to the corresponding author.

## Supplementary Materials

The following supporting information can be downloaded at https://www.mdpi.com/article/10.3390/ijms26083466/s1.
