# Supplementary material for: Apoptotic Caspases-3 and -7 Cleave Extracellular Domains of Membrane-Bound Proteins from MDA-MB-231 Breast Cancer Cells
Source: Int J Mol Sci. 2025 Apr 8;26(8):3466. doi: 10.3390/ijms26083466 (PMC12026882; doi:10.3390/ijms26083466)
Supplement: Supplementary file 1 [file ijms-26-03466-s001.zip › ijms-3440670-supplementary.pdf]

Supplementary material

Article

Apoptotic Caspases-3 and -7 Cleave Extracellular Domains of Membrane-Bound Proteins from MDA-MB-231 Breast Cancer Cells

Eva Vidak [1,2], Matej Vizovišek [1,†], Nežka Kavčič [1,‡], Monika Biasizzo [1], Marko Fonović [1] and Boris Turk [1,3,*]

[1] Department of Biochemistry and Molecular and Structural Biology, Jožef Stefan Institute, Jamova 39, SI-1000 Ljubljana, Slovenia; evidakijs@gmail.com (E.V.); mvizovisek@gmail.com (M.V.); nezkic@gmail.com (N.K.); monika.biasizzo@gmail.com (M.B.); marko.fonovic@ijs.si (M.F.)
[2] Jožef Stefan International Postgraduate School, Jamova cesta 39, SI-1000 Ljubljana, Slovenia
[3] Faculty of Chemistry and Chemical Technology, University of Ljubljana, Vecna pot 113, SI-1000 Ljubljana, Slovenia
* Correspondence: boris.turk@ijs.si
† Current address: Mattackerstrasse 6, 8052 Zürich, Switzerland.
‡ Current address: ICGEB, Padriciano 99, 34149 Trieste, Italy.

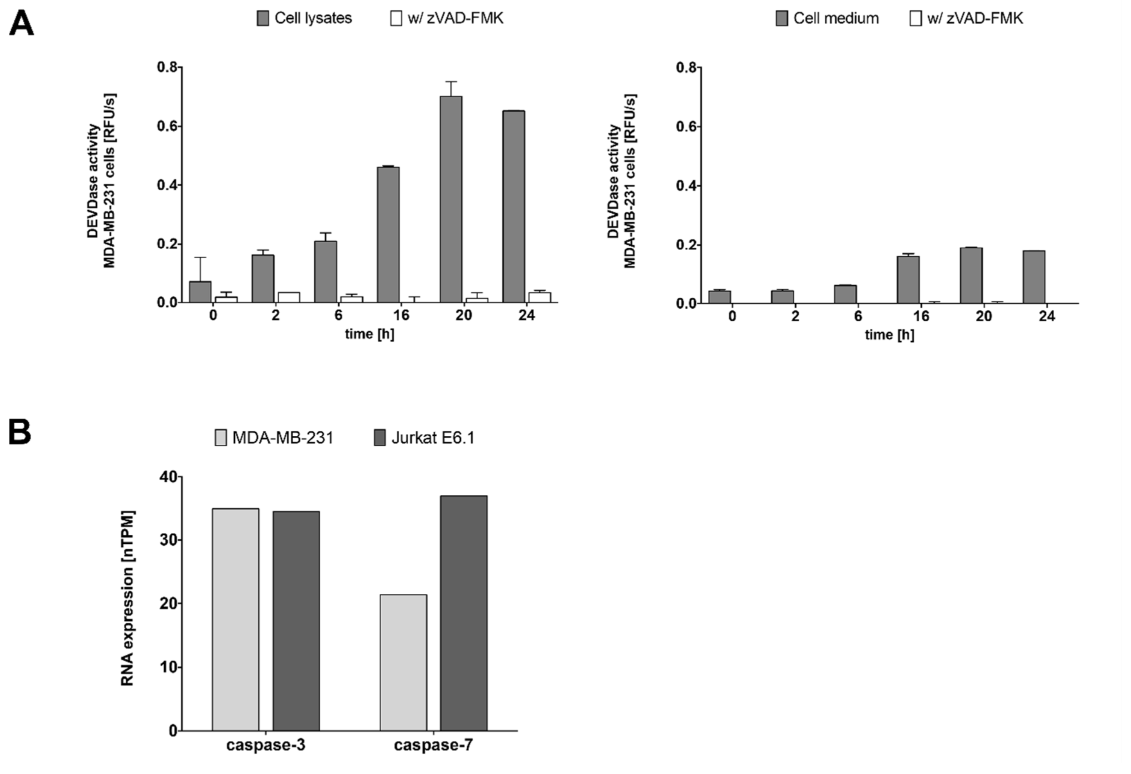

Figure S1. Enzymatic activity of caspases-3 and -7 in lysates and cell media of apoptotic MDA-MB-231 cells and presentation of transcriptomic data from The Humam Protein Atlas. (**A**) DEVDase activity in lysates and cell media from MDA-MB-231 cells treated with 0,5 µM STS at different time points. (**B**) RNA expression levels of both caspases in MDA-MB-231 cells as compared to Jurkat E6.1 cells acquired from The Human Protein Atlas. *nTPM, normalized transcript per million.*

A

pH of concentrated pooled cell medium		
**Without additions**		
**Time point**	**pH without incubation**	**pH after 1h at 37 °C**
0h STS	7.65	7.55
20h STS	7.81	7.68
**With shedding conditions**		
0h STS	7.63	7.57
0h STS + zVAD	7.59	7.56
20h STS	7.75	7.85
20h STS + zVAD	7.73	7.77
**With 10×MES buffer, pH 6.0**		
0h STS	6.28	6.37
0h STS + zVAD	6.45	6.46
20h STS	6.62	6.57
20h STS + zVAD	6.46	6.57
**With 10×HEPES buffer, pH 7.4**		
0h STS	7.28	7.32
0h STS + zVAD	7.32	7.34
20h STS	7.37	7.40
20h STS + zVAD	7.49	7.50

B

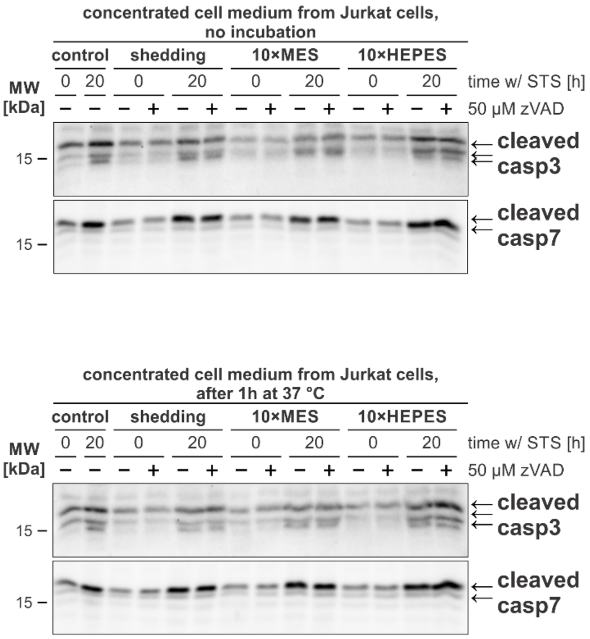

Figure S2. Influence of the concentrated buffers on the pH of pooled concentrated media from Jurkat cells and the detection of caspases-3 and -7 in the media. Concentrated media from Jurkat cells were pooled from several separate experiments (n=11) were used both for pH determination (**A**) and Western blot detection of cleaved caspases-3 and -7 (**B**) both with and without incubation at 37 °C to simulate conditions used for kinetic analysis.

Table S1. List of top 10 proteins identified by MS as potential substrates of caspases-3 and -7. The list is limited to substrates with known association with the membrane and sorted according to the average SCR for treatment with both caspases.

			Caspase-3		Caspase-7		
**Protein name**	**Gene name**	**UniProtKB ID**	**MS/MS count (1µM casp3)**	**SCR**	**MS/MS count (1µM casp7)**	**SCR**	**Average SCR**
Protocadherin Fat 1	FAT1	Q14517	20	21.0	31	32.0	26.5
Receptor-type tyrosine-protein phosphatase gamma	PTPRG	P23470	22	23.0	21	22.0	22.5
Nesprin-2	SYNE2	Q8WXH0	20	21.0	10	11.0	16.0
Neuropilin-1	NRP1	O14786	51	10.4	57	11.6	11.0
Hepatocyte growth factor receptor	MET	P08581	10	11.0	8	9.0	10.0
V-set and immunoglobulin domain-containing protein 8	VSIG8	Q5VU13	0	1.0	17	18.0	9.5
Semaphorin-4B	SEMA4B	Q9NPR2	4	5.0	11	12.0	8.5
Complement decay-accelerating factor	CD55	P08174	9	10.0	6	7.0	8.5
Integrin alpha-6	ITGA6	P23229	10	11.0	4	5.0	8.0
Major prion protein	PRNP	P04156	7	8.0	5	6.0	7.0

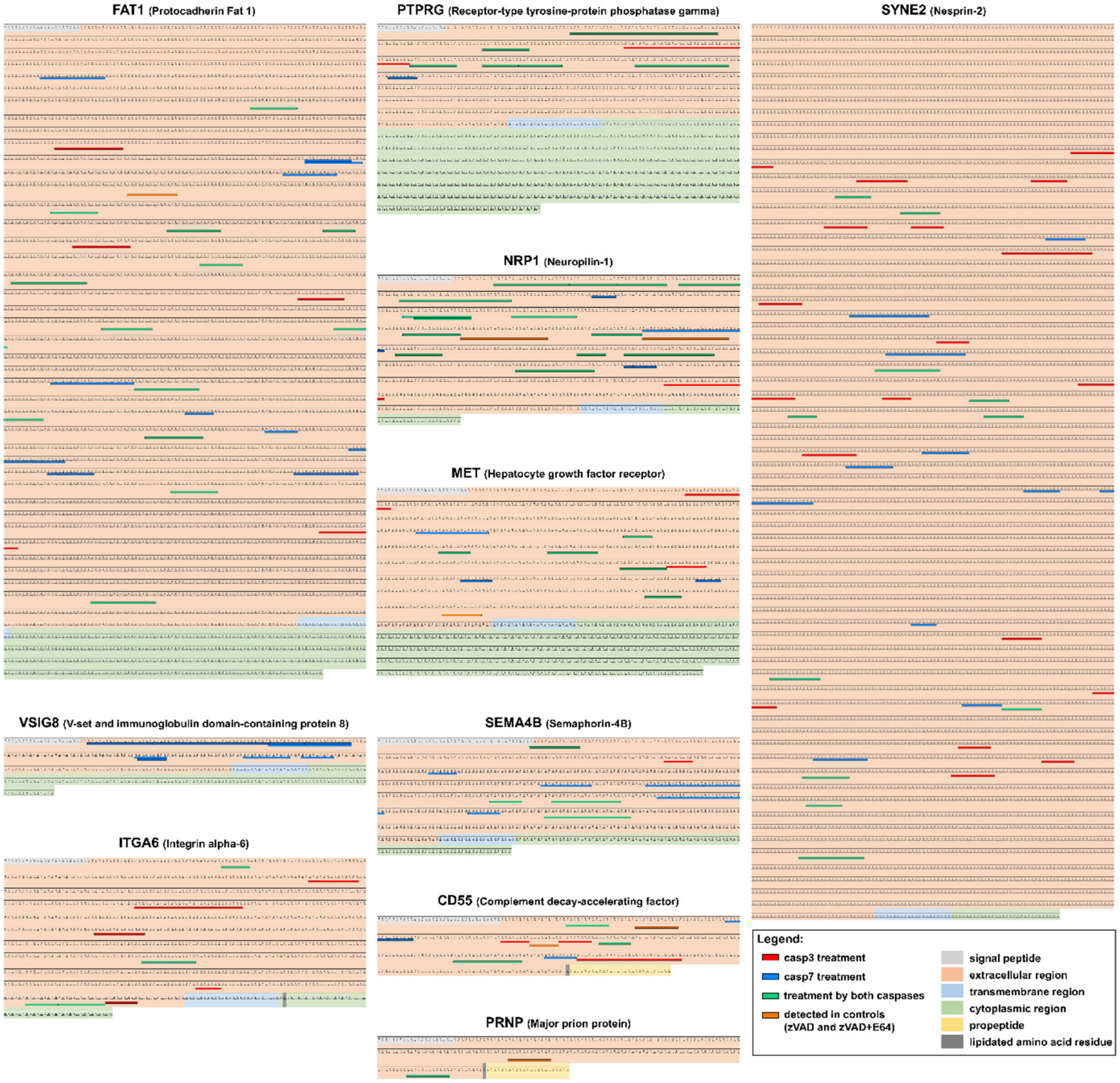

Figure S3. Visualization of peptide coverage for the 10 selected identified substrates of both caspases. Using the Draw Map MS Tool, peptides detected after treatment with caspase-3 (red), caspase-7 (blue), both caspases (green), and those detected in controls containing either only zVAD or both zVAD and E-64 (orange) were fitted to the amino acid sequence of selected substrates acquired from the UniProt KB database.

A

Possible predicted cleavage sites			
**caspase-3 ↓**			
**Position inside the extracellular domain**	**P4-P4' Site**	**Predicted N-fragment size**	**Procleave score**
584	DECD↓DDQA	71,74 kDa	0,878
646	WEHD↓NHVQ	79,40 kDa	0,606
**caspase-7 ↓**			
**Position inside the extracellular domain**	**P4-P4' Site**	**Predicted N-fragment size**	**Procleave score**
584	DECD↓DDQA	71,74 kDa	0,801
646	WEHD↓NHVQ	79,40 kDa	0,641

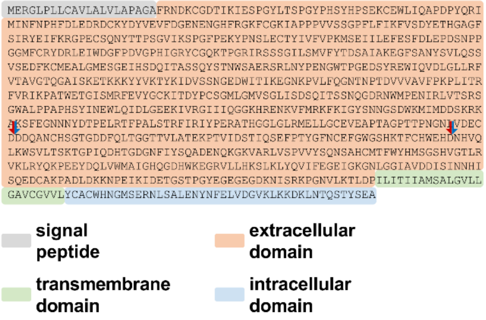

B

Possible predicted cleavage sites			
**caspase-3 ↓**			
**Position inside the extracellular domain**	**P4-P4' Site**	**Predicted N-fragment size**	**Procleave score**
2092	DLED↓GRLG	246,86 kDa	0,975
1895	SMSD↓GASP	223,55 kDa	0,916
**caspase-7 ↓**			
No site matches western blot results			

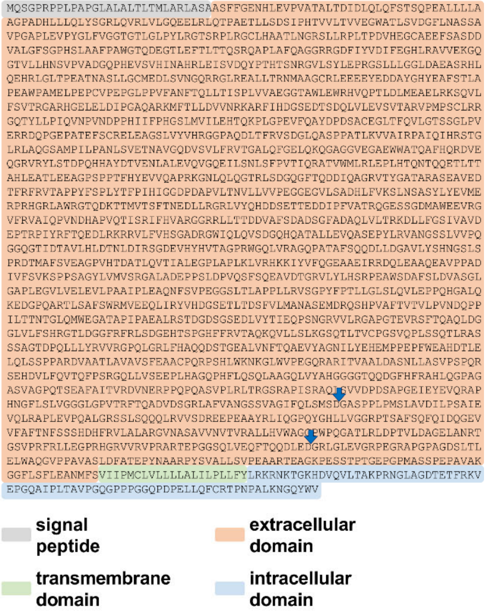

C

Possible predicted cleavage sites			
**caspase-3 ↓**			
**Position inside the extracellular domain**	**P4-P4' Site**	**Predicted N-fragment size**	**Procleave score**
605	SESD↓GHSH	71,06 kDa	0,896
**caspase-7 ↓**			
**Position inside the extracellular domain**	**P4-P4' Site**	**Predicted N-fragment size**	**Procleave score**
605	SESD↓GHSH	71,06 kDa	0,871

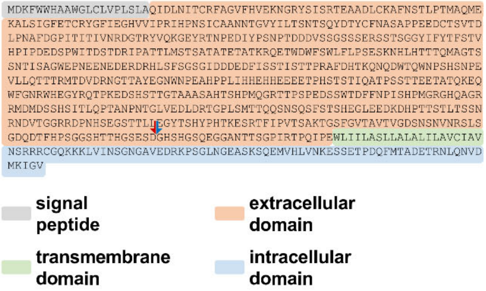

Figure S4. Bioinformatic prediction of caspase-3 and -7 cleavage sites. For prediction, sequences of extracellular domains of selected targets—NRP-1 (**A**), CSPG4 (**B**), and CD44 (**C**)—were analyzed by the Procleave algorithm, and the acquired results were compared to the cleavages detected with Western blot. Cleavage sites with the highest probability score (Procleave score) are indicated with either a blue arrow (caspase-3 cleavage), a red arrow (caspase-7 cleavage), or a two-colored red and blue arrow (predicted cleavage site for both caspases).

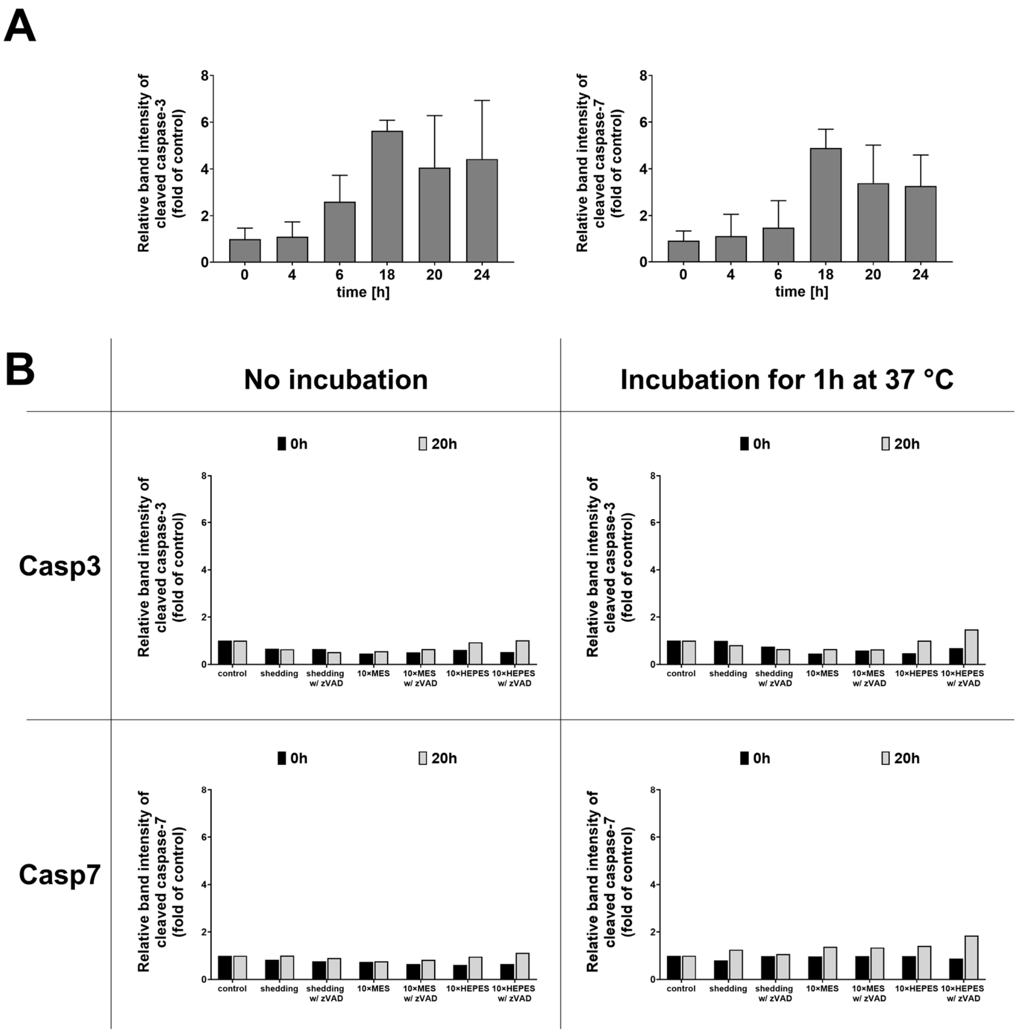

Figure S5. Relative intensity of detected cleaved caspase-3 and caspase-7 by Western blotting in the cell media of STS treated Jurkat E6.1 cells. (A) Relative intensity of observed bands in the cell medium for Western blot shown in Figure 2B in the main article. Intensity is depicted as the fold of control with the control being the untreated (0h STS) cells and calculated for three independent experiments (n=3). (B) Calculated relative intensity of bands observed in pooled concentrated cell media from STS-treated Jurkat E6.1 cells shown in Figure S2B in the Supplementary materials. Relative intensity is shown as the fold of control for each time of exposure to STS (0h STA and 20h STS).

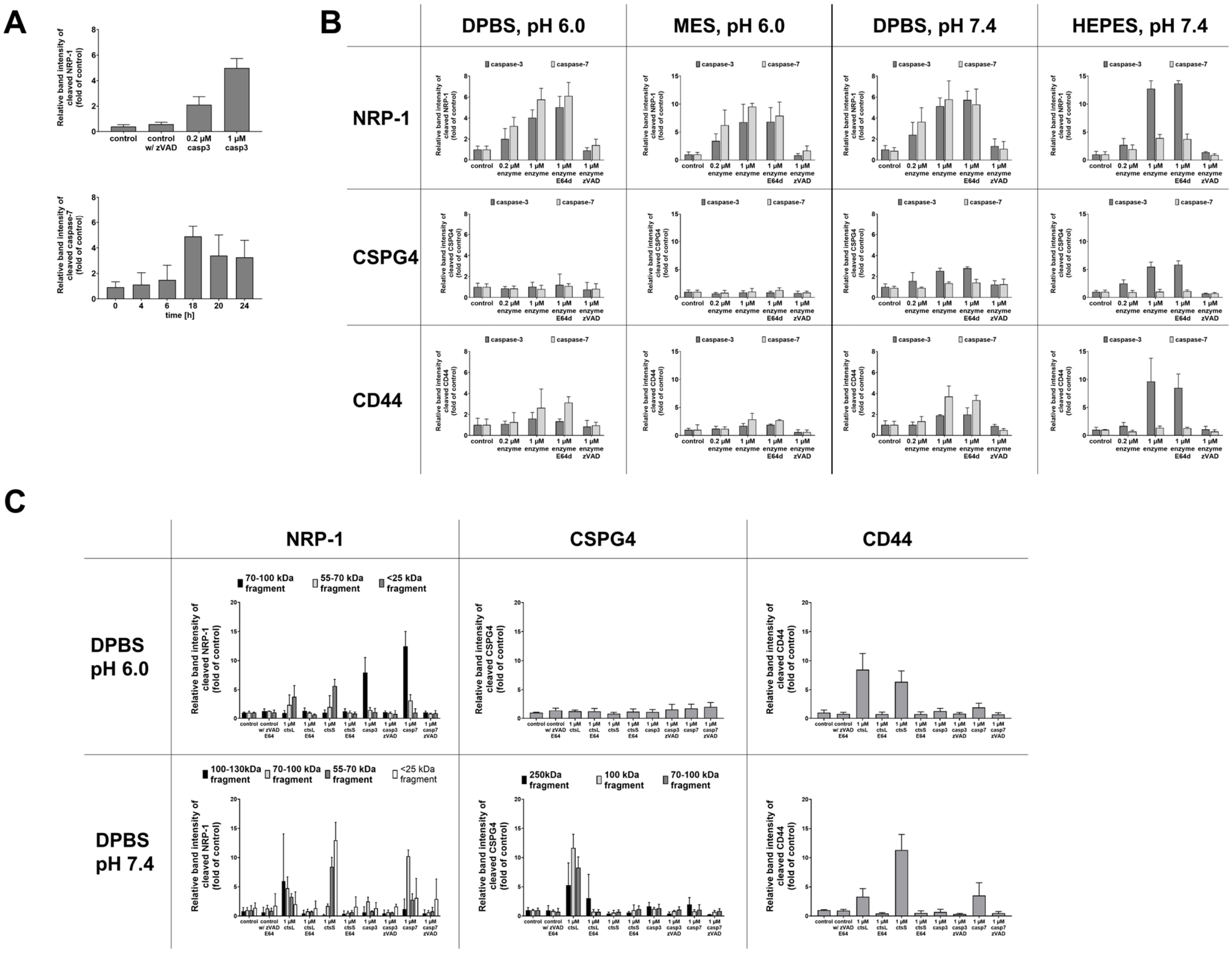

Figure S6. Relative intensity of detected bands for cleaved fragments of selected targets (NRP-1, CSPG4 and CD44) present in the sheddomes after treatment of MDA-MB-231 cells with either solely recombinant apoptotic caspases-3 and -7 or with apoptotic caspases and cysteine cathepsins L and S. The calculated relative intensity of three independent experiments (n=3) is expressed as the fold of control with MDA-MB-231 cells treated with just the buffer (control condition) being used as control. In all cases, the bands observed in the total lysate of MDA-MB-231 cells were excluded from analysis due to different loadings and since they were only used to show the size difference between full-length proteins and cleaved extracellular fragments. Presented relative intensities correspond to Western blots in the main article as follows: (A) Figure 4D, (B) Figure 5, and (C) Figure 6.
